# Surface-Based Falff: A Potential Novel Biomarker for Prediction of Radiation Encephalopathy in Patients With Nasopharyngeal Carcinoma

**DOI:** 10.3389/fnins.2021.692575

**Published:** 2021-07-19

**Authors:** You-ming Zhang, Ya-fei Kang, Jun-jie Zeng, Li Li, Jian-ming Gao, Li-zhi Liu, Liang-rong Shi, Wei-hua Liao

**Affiliations:** ^1^Department of Radiology, Xiangya Hospital, Central South University, Changsha, China; ^2^School of Psychology, Shaanxi Normal University, Shaanxi Provincial Key Research Center of Child Mental and Behavioral Health, Xi'an, China; ^3^Department of Radiology, Hunan Children's Hospital, Changsha, China; ^4^Sun Yat-sen University Cancer Center, State Key Laboratory of Oncology in South China, Collaborative Innovation Center for Cancer Medicine, Guangzhou, China; ^5^Department of Radiation Oncology, Collaborative Innovation Center for Cancer Medicine, State Key Laboratory of Oncology in South China, Sun Yat-sen University Cancer Center, Guangzhou, China

**Keywords:** radiation encephalopathy, nasopharyngeal carcinoma, machine learning, surface-based fALFF, imaging biomarker

## Abstract

Radiation encephalopathy (RE) is an important potential complication in patients with nasopharyngeal carcinoma (NPC) who undergo radiotherapy (RT) that can affect the quality of life. However, a functional imaging biomarker of pre-symptomatic RE has not yet been established. This study aimed to assess radiation-induced gray matter functional alterations and explore fractional amplitude of low-frequency fluctuation (fALFF) as an imaging biomarker for predicting or diagnosing RE in patients with NPC. A total of 60 patients with NPC were examined, 21 in the pre-RT cohort and 39 in the post-RT cohort. Patients in the post-RT cohort were further divided into two subgroups according to the occurrence of RE in follow-up: post-RT _non−RE_ (*n* = 21) and post-RT _REproved_ _infollow−up_ (*n* = 18). Surface-based and volume-based fALFF were used to detect radiation-induced functional alterations. Functional derived features were then adopted to construct a predictive model for the diagnosis of RE. We observed that surface-based fALFF could sensitively detect radiation-induced functional alterations in the intratemporal brain regions (such as the hippocampus and superior temporal gyrus), as well as the extratemporal regions (such as the insula and prefrontal lobe); however, no significant intergroup differences were observed using volume-based fALFF. No significant correlation between fALFF and radiation dose to the ipsilateral temporal lobe was observed. Support vector machine (SVM) analysis revealed that surface-based fALFF in the bilateral superior temporal gyri and left insula exhibited impressive performance (accuracy = 80.49%) in identifying patients likely to develop RE. We conclude that surface-based fALFF may serve as a sensitive imaging biomarker in the prediction of RE.

## Introduction

Nasopharyngeal carcinoma (NPC) is a malignancy that originates from the epithelia of the nasopharynx. It is characterized by a distinct geographical distribution, with the vast majority of confirmed cases occurring in southern China (Chua et al., 2016; Chen et al., 2019). Pathologically, ~95% of NPCs are poorly differentiated squamous cell carcinomas that are sensitive to radiotherapy (RT) (Zhang et al., 2013). However, RT can also damage normal tissues, which can result in radiation-related complications such as radiation encephalopathy (RE). The reported incidence of RE after RT for NPC ranges between 3.2 and 12.9% (Wang et al., 2019). It manifests a variety of neuropsychiatric symptoms that can seriously affect the quality of life of the patient, such as headache, cognitive impairment, depression, and sometimes epilepsy. A deeper understanding of RE pathogenesis and the ability to predict RE in individual patients is of great clinical significance.

Currently, RE diagnosis mainly depends on conventional CT and MRI. The typical imaging finding is radiation-induced brain necrosis, which indicates the irreversibility of this disease. New techniques are required for early diagnosis and prevention. Recent studies using magnetic resonance spectroscopy (MRS) and diffusion tensor imaging (DTI) have provided evidence of abnormal metabolites and abnormal cerebral white matter microstructure, respectively (Chen et al., 2014; Duan et al., 2016). However, the choice of the region of interest for use in MRS is subjective and nonreproducible, and DTI cannot detect alterations in gray matter (GM). Recently, voxel-based morphometry (VBM) and surface-based morphometry (SBM) have been used to examine radiation-induced alterations in volume, cortical thickness, and cortical surface area in normal-appearing GM following RT in patients with NPC (Lv et al., 2014; Lin et al., 2017; Zhang et al., 2018). However, these techniques do not provide hemodynamic information.

Resting-state functional MRI (rsfMRI) allows the acquisition of hemodynamic information and can provide new insights into disease pathophysiology (Dai et al., 2015). The metrics of regional homogeneity (ReHo) and amplitude of low-frequency fluctuation (ALFF) reflect regional spontaneous neuronal activity in the blood oxygenation-level dependent (BOLD) signal and have been used as powerful indicators in the assessment of various central nervous system (CNS) diseases (Zang et al., 2007; Wu et al., 2009; Mankinen et al., 2011; Liu et al., 2012, 2013; An et al., 2013; Li et al., 2016; Wang et al., 2016). Several rsfMRI studies using ReHo and ALFF analysis have reported that patients with NPC treated with RT show altered regional spontaneous neuronal activity not only in the temporal lobes but also in other regions, such as the posterior cingulate cortex (PCC), the precuneus, the calcarine sulcus, and the lingual gyrus (Ding et al., 2018; Zhang et al., 2020a). Because ALFF is sensitive to physiological noise, fractional ALFF (fALFF) (the ratio of the low-frequency power spectrum [0.01–0.08 Hz] to that of the entire frequency range) has been proposed as a metric that significantly suppresses the non-specific signal components in the cisterns (Zou et al., 2008). However, to the best of our knowledge, fALFF has not been examined in patients with NPC following RT. Considering the frequently reported three-dimensional (3D) volume-based and surface-based radiation-induced structural changes in the GM of patients with NPC (Lv et al., 2014; Lin et al., 2017; Zhang et al., 2018), a comprehensive functional assessment of this disease should simultaneously investigate GM alterations from both 3D and two-dimensional (2D) perspectives.

In this study, we first conducted volume-based and surface-based fALFF analyses to characterize GM functional alterations in patients with NPC following RT by comparing alterations between patients in the pre- and post-RT cohorts. Second, voxel/vertex-wise correlation analyses were performed to examine the relationship between fALFF and the maximum dosage of radiotherapy (MDRT) to the ipsilateral temporal lobe. Third, we applied a machine learning strategy to evaluate fALFF as a predictor of RE. Given that a surface-based functional metric has higher test–retest reliability and is more specific to the intrinsic functional organization of the cortical mantle (Li et al., 2014; Jiang and Zuo, 2016; Zhang B. et al., 2019), we hypothesized that surface-based fALFF is an effective biomarker that reflects the neuropathologic alterations underlying RE and can identify patients who are likely to experience RE in the pre-symptomatic stage.

## Materials and Methods

### Subjects

**Figure 1:**
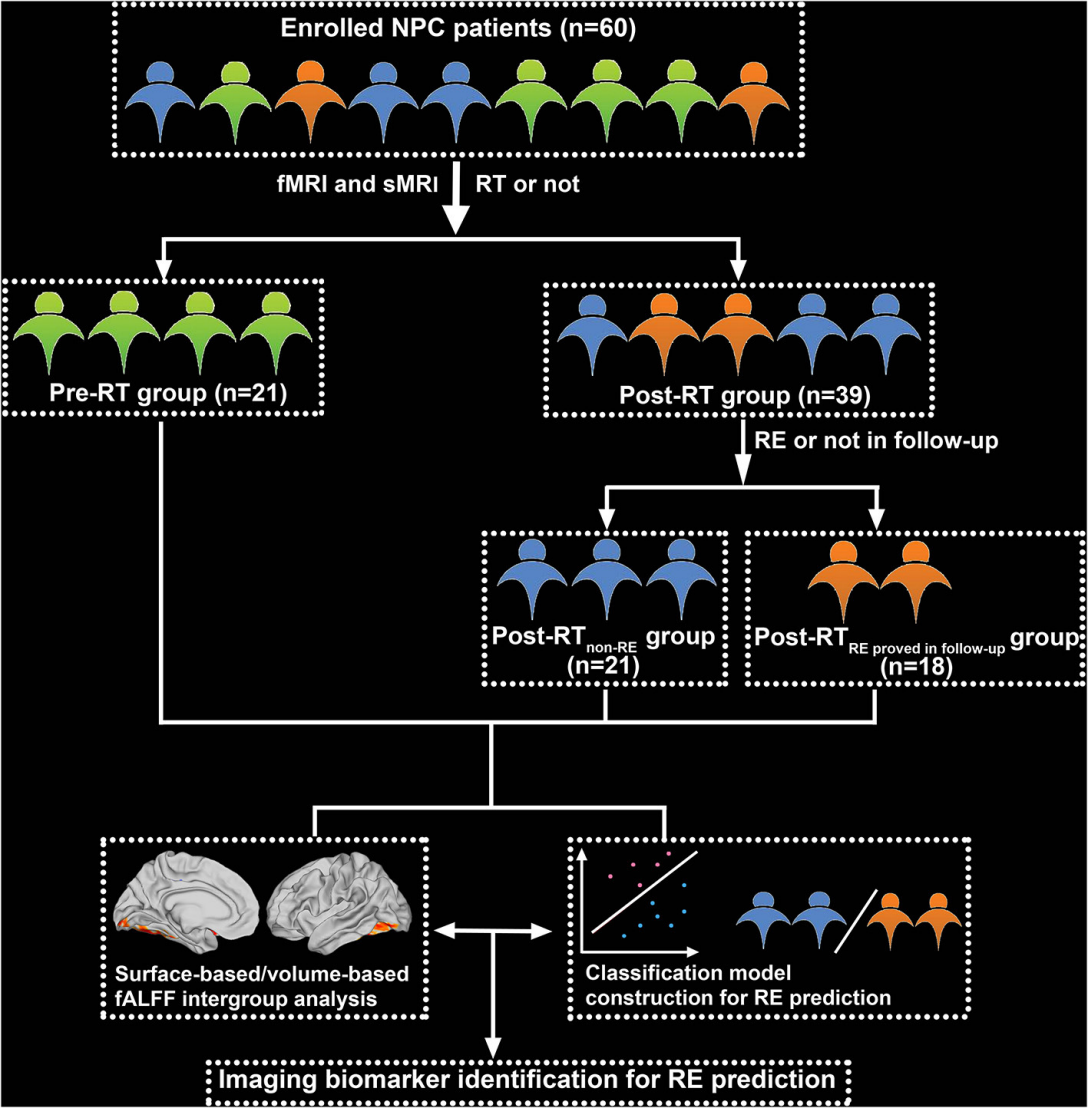
Flowchart for grouping and analysis of the enrolled patients with nasopharyngeal carcinoma (NPC). fMRI, functional MRI; sMRI, structural MRI; RT, radiotherapy; RE, radiation encephalopathy.

The study population comprised 60 patients with NPC, 21 in the pre-RT cohort, and 39 in the post-RT cohort. Patients in the post-RT cohort were further divided into two subgroups according to the occurrence of RE in follow-up: post-RT _non−RE_ (*n* = 21) and post-RT _RE proved_ _in follow−up_ (*n* = 18). The diagnostic criteria and MRI findings for RE are shown in Supplementary Material. The study flow chart is illustrated in Figure 1.

Nasopharyngeal carcinoma clinical staging was performed according to the 7th edition of the UICC/AJCC TNM (2009). Patients in the post-RT cohort received intensity-modulated radiation therapy (IMRT) and 2D conventional radiotherapy (2D-CRT); details are presented in Supplementary Material. For patients with stage IIb to IVa–b of the disease, concurrent chemoradiotherapy with/without neoadjuvant/adjuvant chemotherapy was recommended (Table 1); details are described in Supplementary Material. To minimize the confounding effect of chemotherapy on surface-based fALFF alterations, we tried our best to make the between-group clinical stages of enrolled patients with NPC balanced (Table 1) and to make the between-group chemotherapy regimens standard and unified (Zhang et al., 2018). Patient inclusion criteria were as follows: (1) pathological confirmation of NPC; (2) normal-appearing brain on conventional MRI sequences; (3) Karnofsky Performance Scale score > 80; (4) age < 65 years, and (5) right-handedness (Zhang et al., 2018, 2020a). We excluded patients with NPC who had brain invasion, had brain atrophy or tumor, a history of psychiatric or neurological disease or intracranial surgery, and had other substantial intracranial disease or MRI contraindications (Zhang et al., 2018, 2020a).

**Table 1:** Clinical parameters.

**Clinical features**	**Pre-RT group (*n* = 21)**	**Post-RT_**non-RE**_ group (*n* = 21)**	**Post-RT_**RE proved in follow-up**_ group (*n* = 18)**	***p* value**
**Age (years) mean** **±** **SD**	45.10 ± 9.17	42.95 ± 11.56	45.17 ± 9.52	0.73
**Sex**, ***n***
Male	17 (28.33)	17 (28.33)	14 (23.33)	
Female	4 (6.67)	4 (6.67)	4 (6.67)	0.96
**Clinical staging**
I/II, *n*	5 (8.33)	4 (6.67)	6 (10.00)	
III/IV, *n*	16 (26.67)	17 (28.33)	12 (20.00)	0.58
**Time intervals between RT and fMRI examinations (month)**		13.05 ± 13.88	18.44 ± 17.38	0.29
**RT technology**
IMRT, *n*	NA	15 (38.46)	12 (30.77)	
2D-CRT, *n*	NA	6 (15.38)	6 (15.38)	0.75
**Maximum dosage of RT for temporal lobes (Gy)**
Left	NA	69.21 ± 5.12^#^	64.41 ± 7.71^*^	0.09
Right	NA	67.38 ± 6.36^#^	66.54 ± 9.06^*^	0.80
**The location of RE**
Left, *n*	NA	NA	8 (44.44)	
Right, *n*	NA	NA	2 (11.12)	
Bilateral, *n*	NA	NA	8 (44.44)	NA

*RT, radiotherapy; RE, radiation encephalopathy; SD, standard deviation; fMRI, functional magnetic resonance imaging; IMRT, intensity-modulated radiation therapy*.

*2D-CRT, conventional two-dimensional radiotherapy; NA denotes not available*;

# *denotes radiation dose of six patients were not available*;

* *denotes radiation dose of ten patients were not available. Data in parentheses are percentages*.

### MRI Acquisition

Data from MRI were obtained with a Magnetom Tim Trio 3T scanner (Seimens, Munich, Germany). Three-dimensional T1-weighted magnetization prepared rapid acquisition gradient echo (MPRAGE) and BOLD sequences were used for data analysis. Three-dimensional T1 sequence parameters were as follows (Zhang et al., 2018, 2020a): voxel size = 1.0 × 1.0 × 1.0 mm, field of view (FOV) = 256 × 256 mm, echo time (TE) = 2.98 ms, repetition time (TR) = 2,300 ms, thickness/gap = 1.0/0 mm, matrix size = 256 × 256, flip angle = 9°, and 176 sagittal slices. BOLD sequence parameters were as follows: total time points = 240, TR = 2400 ms, matrix size = 64 × 64, flip angle = 90°, TE = 30 ms, FOV = 230 × 230 mm, and 40 axial slices. The patients were instructed to remain awake and calm with eyes closed during rsfMRI scanning.

### fMRI Data Preprocessing

All imaging data were preprocessed using DPABI Surf 5.0 and SPM8 based on Matlab 2016b. The DPABISurf pipeline (http://rfmri.org/DPABISurf) provides a data-preprocessing platform for both brain structure and function. The main structural image preprocessing steps included intensity correction, skull stripping, spatial normalization, brain tissue segmentation, and surface reconstruction (Dale et al., 1999; Yan et al., 2016). The functional imaging preprocessing steps were as follows: (1) brain mask generation; (2) head motion estimation; (3) slice timing; (4) multiple linear regression (Yan et al., 2013b; Zuo et al., 2013); and (5) alignment of spatial correspondences to individual structural images using a boundary-based registration (BBR) algorithm (Zuo et al., 2013). The preprocessing steps for volume-based fALFF are shown in Supplementary Material.

Considering that rsfMRI data are sensitive to head motion (Van Dijk et al., 2012; Yan et al., 2013a), we used the following measures to reduce motion effects: (1) A participant was rejected if the maximum translational displacement (x, y, or z directions) was >2.5 mm or the maximum rotation was >2.5°, and (2) vertex-/voxel-specific frame-wise displacement measures were regressed out as nuisance covariates. The global signal was regressed out.

### Falff Analysis

After data preprocessing, the time series for each vertex/voxel was temporally bandpass filtered (0.01–0.1 Hz) and linearly detrended to reduce physiological high-frequency noise and low-frequency drift. Calculation of fALFF was performed as previously described (Zou et al., 2008). A 2D/3D fALFF map was obtained for each participant, which was then divided by the mean 2D/3D fALFF value of the whole brain to facilitate comparisons between participants.

### SVM Analysis

Support vector machine (SVM) classifier was employed for classification and was implemented using the LIBSVM toolbox (http://www.csie.ntu.edu.tw/~cjlin/libsvm). A linear kernel SVM was adopted to reduce the risk of overfitting. The detailed information was described as follows:

### Cross-Validation

Owing to the limited number of subjects, leave-one-out-cross-validation (LOOCV) was adopted in this study to estimate the SVM classification and its prediction accuracy for it could give the most unbiased estimate of test error (Hastie et al., 2001; Vergun et al., 2013). In each fold, one subject was used as the test set, and the others were used as the training set for feature selection (see below) and classification. The procedure was repeated until every enrolled subject was used as the test set. Then, we calculated the classification accuracy (Shi and Liu, 2020).

### Feature Selection

The averaged surfaced-based fALFF values of 360 regions based on the HCP_MMP 1.0 template (Glasser et al., 2016) were obtained as input features in the subsequent machine learning analysis. During each SVM LOOCV fold, two-sample *t*-tests on the post-RT _non−RE_ and post-RT _RE proved in follow−up_ groups of the training set were performed to select the best features for improving the efficiency of classification. The two-sample *t*-tests were conducted at a series of *p*-values, which ranged from 0.001 to 0.5 with a step length of 0.001. Specifically, at each *p*-value threshold, two-sample *t*-tests on the training set were used to select cortical brain regions with intergroup differences of 2D fALFF as features, which were successively added into the classifier for training. The trained classification model was then used to make a prediction in the test set, and the classification accuracy was finally calculated. Under each *p* value threshold, the cortical regions of 2D fALFF existing in all cross-validation folds were determined as final selected features of interest for subsequent model explanations.

## Statistical Analysis

### Clinical Data Analysis

Normally distributed quantitative clinical data are presented as means with SD; non-normally distributed data are presented as medians with interquartile range. Qualitative data are presented as frequencies. Intergroup differences in sex, clinical stage, and radiation therapy technique were compared using the chi-squared test; age difference was compared using a one-way ANOVA. For the post-RT subgroups, intergroup differences in the time interval between RT and fMRI examination and maximum temporal lobe RT dosage were compared using the two-sample *t*-test. *p* < 0.05 was considered significant.

### Two-Dimensional/Three-Dimensional Falff Analysis

To assess intergroup differences in regional GM neuronal activity, all vertex- or voxel-wise contrasts of the filtered and smoothed mean fALFF maps were performed using the statistical analysis module in the DPABI toolbox (http://rfmri.org/dpabi). Specifically, the two-sample *t*-test was performed to detect intergroup differences in 2D and 3D fALFF using pairwise comparisons; sex, age, and vertex-/voxel-specific frame-wise displacement were considered nuisance covariates. Considering the small sample size in each group, we used a permutation test (the number of permutations = 5,000) with a threshold-free cluster enhancement (TFCE, family-wise error [FWE] rate < 0.05, two-tailed) (namely, PT-TFCE-FWE, a strict and reliable multiple comparison correction strategy) to obtain the brain regions with inter-group significant differences (Chen et al., 2018; Jiang et al., 2019). To examine the relationships between 3D/2D fALFF changes and radiation dose to the ipsilateral temporal lobe, we performed Pearson's correlation using 3D/2D fALFF values from voxels/vertices in areas showing intergroup differences, and the maximum radiation dose administered. This analysis was performed using SPSS software version 18.0 (IBM Corp., Armonk, NY, USA). *p* < 0.05 was considered significant.

## Results

### Clinical Data

Forty-eight patients were male and 12 were female. The median age was 44.37 years (range, 14–63). The tumor stage ranged from T1N0M0 to T4N2M0. No significant differences were observed between the pre-RT, post-RT _non−RE_, and post-RT _RE proved in follow−up_ groups with respect to sex (*p* = 0.96), age (*p* = 0.73), and clinical stage (*p* = 0.58). In the post-RT group, no significant differences were observed between the post-RT _non−RE_ and post-RT _RE_ _proved in follow−up_ subgroups with respect to radiation therapy technique (*p* = 0.75), the time interval between RT and fMRI examination (*p* = 0.29), and MDRT to the left (*p* = 0.09) and right (*p* = 0.80) temporal lobes. In the post-RT _RE proved in follow−up_ group, the location of RE was the right temporal lobe in two patients, left temporal lobe in eight, and bilateral temporal lobes in eight (Table 1).

### Surface-Based Falff Analysis

**Figure 2:**
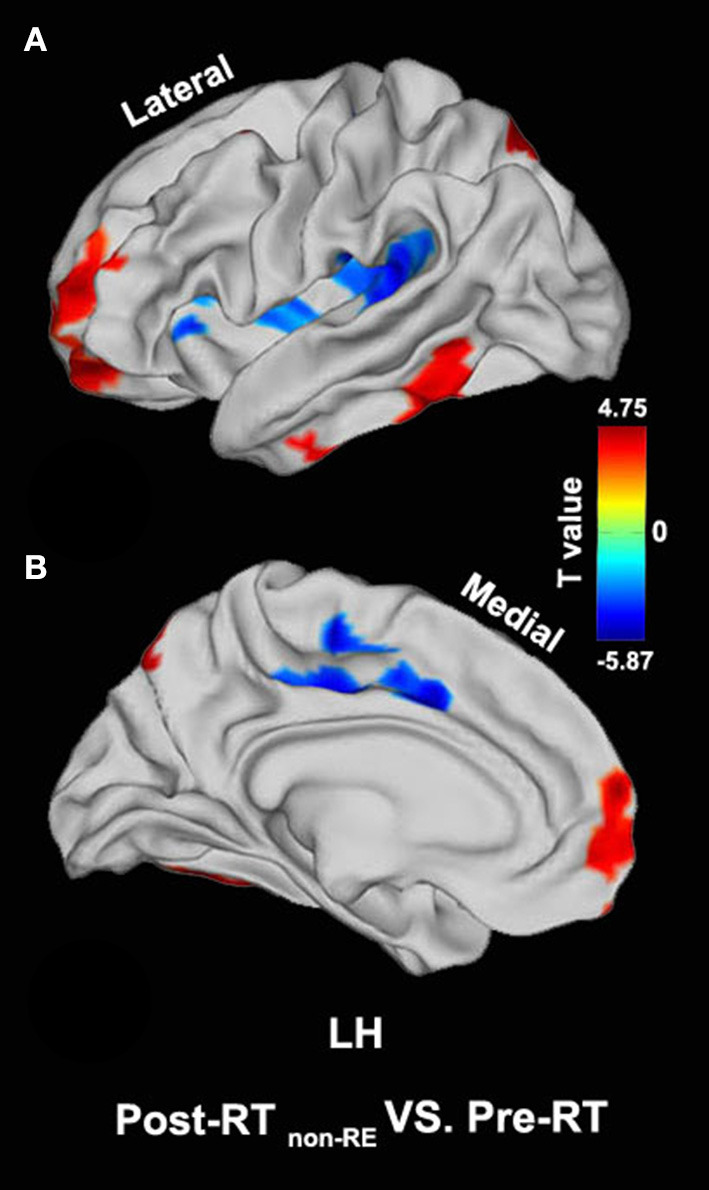
Between-group differences in surface-based fractional amplitude of low-frequency fluctuation (fALFF) [pre- radiotherapy (RT) versus post-RT _non−RE_]. Compared with the pre-RT group, patients in the post-RT _non−RE_ group showed a significantly decreased fALFF in the left paracentral lobule, the cingulate cortex, and the left insula, as well as a significantly increased fALFF in the left inferior temporal gyrus (ITG) and the left prefrontal lobe (*p* < 0.05, PT-TFCE-FWE corrected).

**Figure 3:**
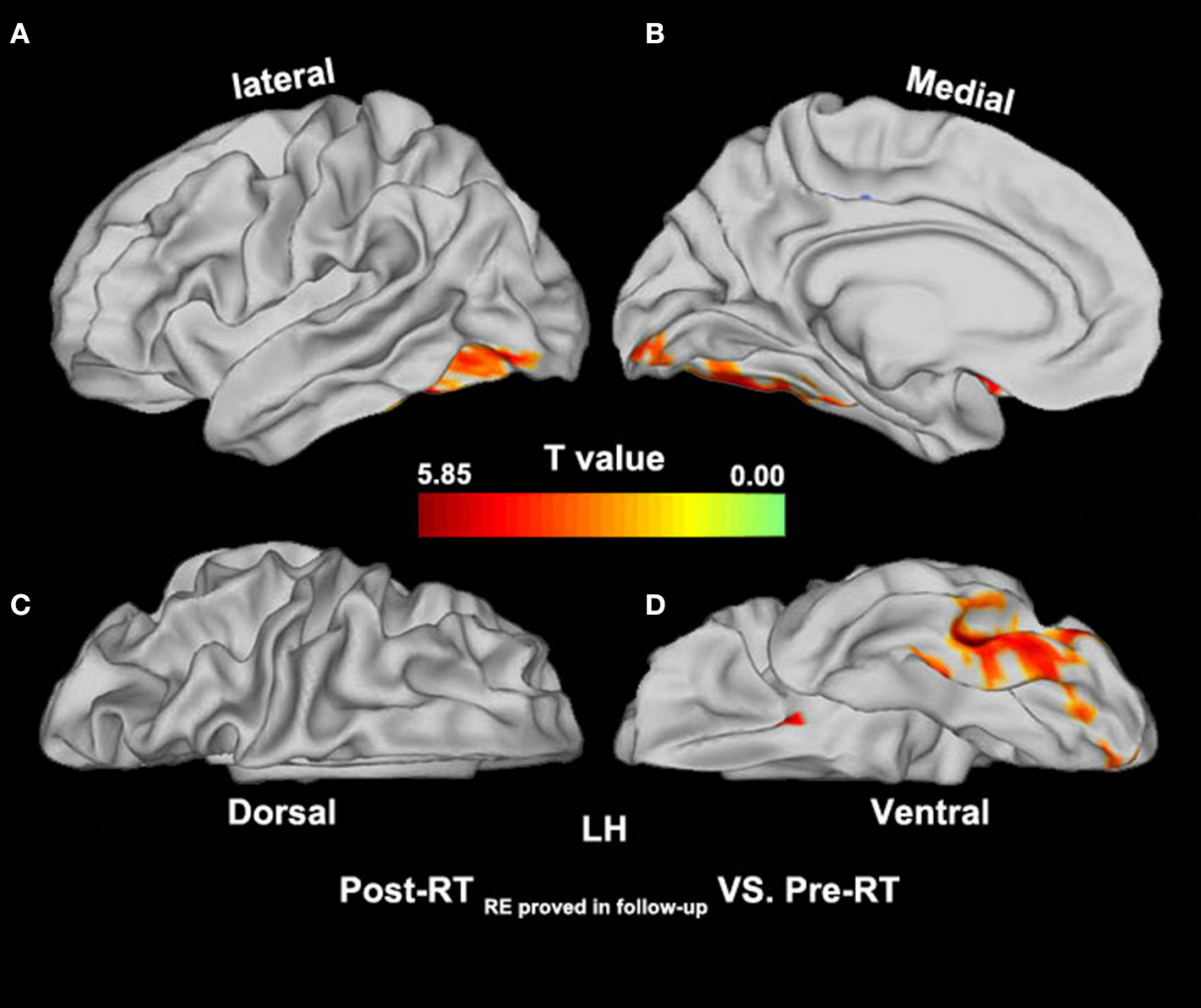
Between-group differences in surface-based fALFF (pre-RT versus post-RT _RE proved in follow−up_). Compared with the pre-RT group, patients in the post-RT _RE proved in follow−up_ group showed significantly increased fALFF in the left mesial temporal lobe, including the hippocampus and the parahippocampal gyrus (*p* < 0.05, PT-TFCE-FWE corrected).

**Figure 4:**
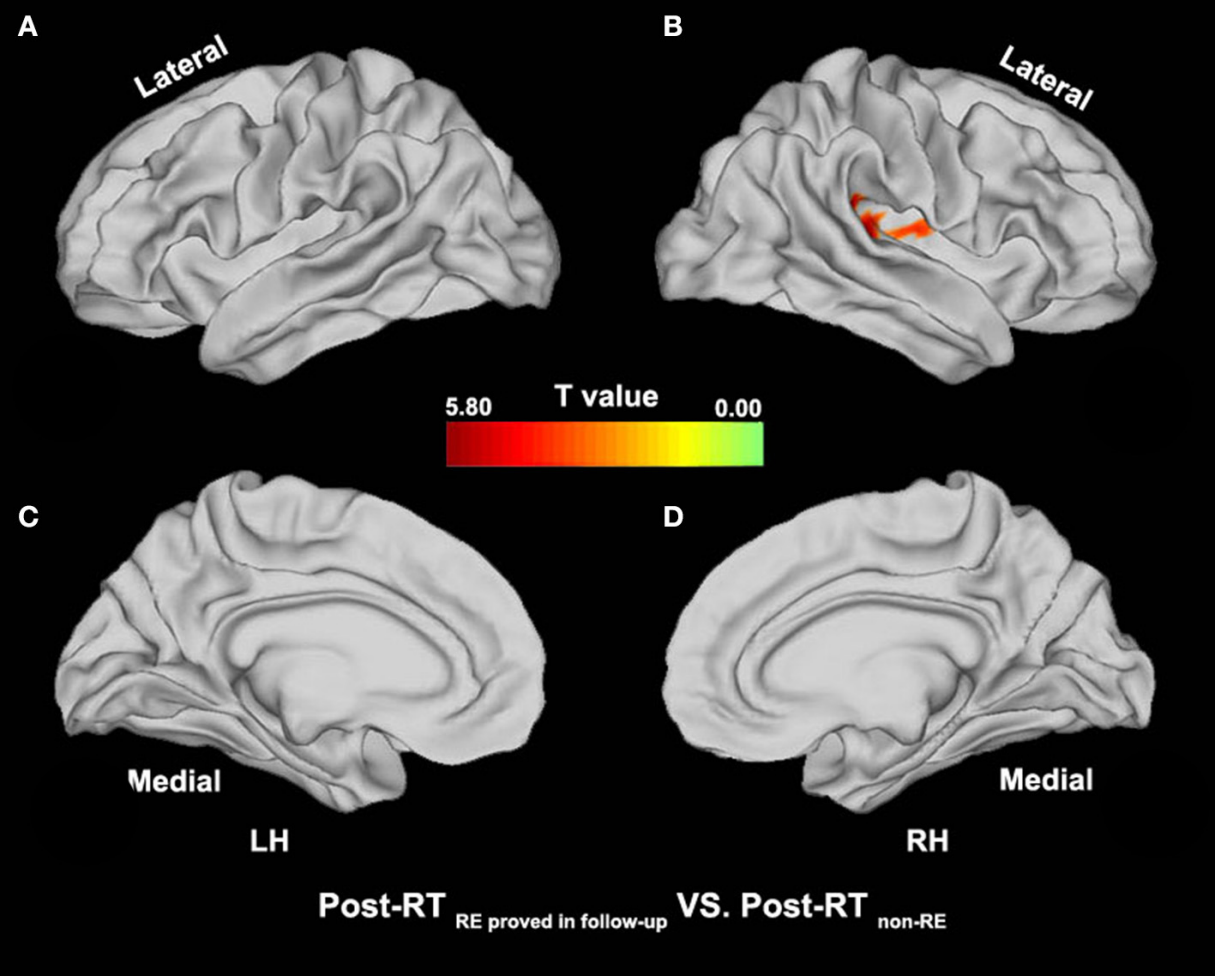
Between-group differences in surface-based fALFF (post-RT _non−RE_ versus post-RT _RE proved in follow−up_). Compared with the Post-RT _non−RE_ group, patients in the Post-RT _RE proved infollow−up_ group showed significantly increased fALFF in the right insula and the right superior temporal gyrus (STG) (*p* < 0.05, PT-TFCE-FWE corrected).

Compared with the pre-RT group, patients in the post-RT _non−RE_ group exhibited a significantly decreased fALFF in the left paracentral lobule, cingulate cortex, and left insula (extending to the adjacent supramarginal gyrus) (*p* < 0.05, PT-TFCE-FWE correction; Figure 2). Furthermore, these patients also showed a significantly increased fALFF in the left inferior temporal gyrus (ITG) and the left prefrontal lobe, including the dorsolateral prefrontal cortex (DLPFC) and the mesial prefrontal cortex (MPFC) *(p* < 0.05, PT-TFCE-FWE correction; Figure 2). Compared with the pre-RT group, patients in the post-RT _RE proved in follow−up_ group showed a significantly increased fALFF in the left mesial temporal lobe, including the hippocampus and the parahippocampal gyrus (*p* < 0.05, PT-TFCE-FWE correction; Figure 3). Moreover, compared with the post-RT _non−RE_ group, patients in the post-RT _RE proved in follow−up_ group showed a significantly increased fALFF in the right insula and right superior temporal gyrus (STG) (*p* < 0.05, PT-TFCE-FWE correction; Figure 4). Detailed information for brain regions with inter-group differences could be found in Supplementary Tables 1–3.

### Volume-Based Falff Analysis

No significant intergroup differences were observed in the pairwise group combinations.

### Correlation Analysis

In the post-RT group (including the post-RT _non−RE_ and post-RT _RE proved in follow−up_ subgroups), no significant correlation was observed between 2D/3D fALFF and MDRT to the ipsilateral temporal lobe.

### SVM Analysis

**Figure 5:**
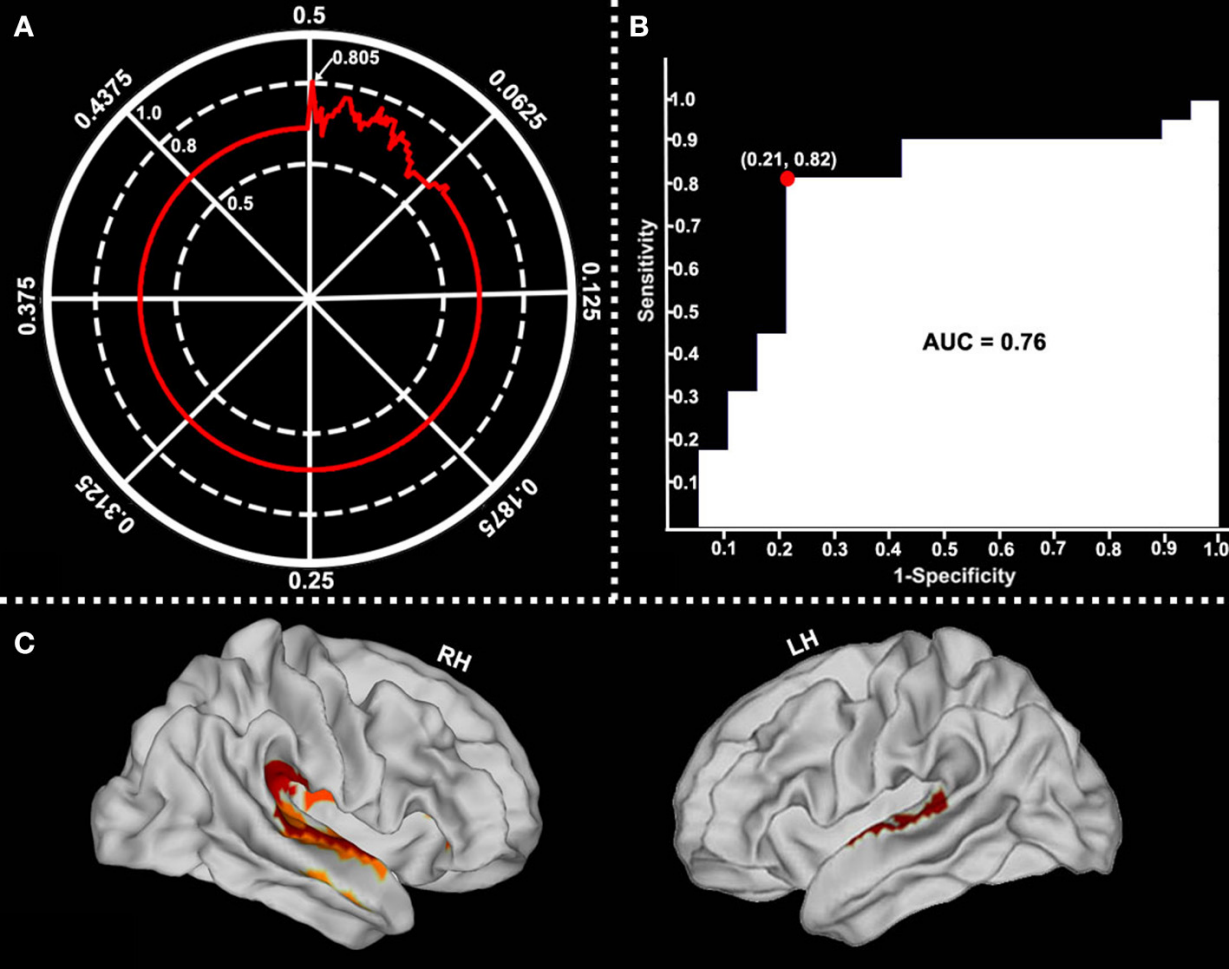
Classification performance of surface-based fALFF at different *p*-value thresholds. Surface-based fALFF in six selected brain regions differentiated by RE from those which were not with a peak accuracy of 80.49% (when *p* = 0.002) (**A**, arrow). The area under the curve (AUC), sensitivity, and specificity were 0.76, 81.82, and 78.95%, respectively (**B**). The main involved brain regions were bilateral STG, the right supramarginal gyrus, and the right insular (**C**).

After comparing the obtained series of prediction accuracies at each of *p-*value threshold *via* the SVM LOOCV method, we observed that, at *p-*value of 0.002, surface-based fALFF in six of the selected cortical brain regions (namely, retro-insular cortex, insular granular complex, parabelt complex, lateral belt complex, auditory 4 complex in the right hemisphere, and lateral belt complex in the left hemisphere, mainly located in the bilateral STG, right supramarginal gyrus, and right insula) differentiated patients with and without RE with a peak accuracy of up to 80.5% (Figures 5A,B). The area under the curve (AUC), accuracy, sensitivity, and specificity of the SVM model were 0.76, 80.49, 81.82, and 78.95%, respectively (Figure 5C).

## Discussion

To the best of our knowledge, this is the first study to use both surface-based and volume-based fALFF to examine the effects of RT on spontaneous neuronal activity in the normal-appearing GM of patients with NPC, which were then used to construct a classification model for identifying patients likely to develop RE. The main findings of this study are as follows: First, surface-based fALFF significantly differed between the study groups in brain regions both inside (such as the bilateral temporal lobes) and outside (such as the bilateral insular cortex and left prefrontal lobe) the radiation field, indicating that radiation-induced brain injury may be a multisystem disorder that involves intratemporal and extratemporal lobe regions. Second, surface-based fALFF (rather than volume-based) was able to detect radiation-induced functional alterations in normal-appearing GM in patients in the post-RT group, suggesting that surface-based fALFF may be a novel imaging biomarker that reflects radiation-induced brain injury pathological processes. Furthermore, surface-based fALFF of the left insula and both superior temporal gyri showed impressive classification performance in the proposed model, which suggests that surface-based fALFF may have an important role in the early diagnosis of RE. These findings may contribute to a better understanding of the potential neural mechanisms involved in RE.

### Intratemporal and Extratemporal Lobe Alterations in Surface-Based Falff

We observed increased 2D fALFF in brain regions within the temporal lobe, including the left ITG, left mesial temporal lobe (including the hippocampus and parahippocampal gyrus), and right STG in the comparison of pairwise group combinations. These findings are consistent with previous neuroimaging studies that reported decreased cortical thickness, increased cortical surface and ReHo in the ITG (Lin et al., 2017; Zhang et al., 2018, 2020a), decreased local gyrification index and local GM volume in the STG (Lv et al., 2014; Zhang B. et al., 2019), and decreased ReHo in the parahippocampal gyrus (Zhang et al., 2020a). The ITG and STG are responsible for speech, language, hearing, and visual perception (Ungerleider and Haxby, 1994; Rajarethinam et al., 2000; Tomasi and Volkow, 2012; Zhang et al., 2018). A decline in both visual and auditory function and language impairment are common clinical symptoms in patients with RT-induced injury (Hu et al., 2003; Hsiao et al., 2010). Our finding of functional alteration in the ITG provides further explanation for the visual, auditory, and verbal functional impairments seen in patients with NPC after RT. Considering that the mesial temporal lobe is crucial for spatial and temporal memory (Squire et al., 2004), altered 2D fALFF in this region is suggestive of cognitive impairment, such as poor short-term memory (Hsiao et al., 2010; Tang et al., 2012). This should be investigated in the future using clinical neuropsychological assessment in conjunction with rsfMRI.

When sketching a nasopharyngeal target, the inclusion of the adjacent temporal lobe in the radiation field cannot be avoided. In particular, the mesial temporal lobe and temporal pole generally receive an intense radiation dose. We speculate that three possible factors, namely, vascular events, tissue sensitivity to radiation exposure, and localized inflammatory reaction, may be responsible for the observed temporal lobe alterations in 2D fALFF. As shown in previous studies, the microcirculatory function is inhibited in irradiated brain areas (Crossen et al., 1994; Sundgren and Cao, 2009; Greene-Schloesser et al., 2012), which induces abnormal blood flow and blood volume, leading to a mismatch between blood oxygen supply and consumption (Fox et al., 1988; Harris et al., 2011). Furthermore, evidence from animal studies has shown that the granule cell layer in the hippocampus is vulnerable because of its high sensitivity to radiation (Monje and Palmer, 2003). Therefore, irradiation of the mesial temporal lobe may induce abnormal brain activity in this region. Moreover, an inflammatory reaction triggered by a series of pro-inflammatory cytokines (such as interleukin-6 and interleukin-1β) released by activated glial cells may be the endogenous substrate that underlies the observed change in 2D fALFF of the temporal lobe.

Our finding of alterations in surface-based fALFF in extratemporal brain regions (such as the prefrontal lobe, the posterior parietal area, the cingulate cortex, and the insula) is of particular interest. The prefrontal lobe and the posterior parietal area are core components of the executive control network (ECN), whereas the cingulate cortex and the insula constitute the salience network (SN) (Seeley et al., 2007). The SN is involved in processing various forms of salient stimuli, such as metabolic stress, social rejection, or pain (Seeley et al., 2007; Zhang et al., 2020b). In contrast, the ECN is equipped to operate on identified salience by weighing against background homeostatic demands, shifting conditions, and context (Seeley et al., 2007). In other words, energy consumption is higher in the ECN when the salient stimuli screening ability of the SN is impaired because it must deal with superfluous input. Therefore, the observed abnormal increase and decrease in 2D fALFF in the ECN and SN, respectively, may occur as a result of network-level functional differentiation and re-organization when various salient stimuli, such as radiation exposure, pain, and social discrimination, are processed. However, the exact cause of this phenomenon remains unclear and requires further investigation. The fact that patients in the post-RT group showed decreased 2D fALFF in the paracentral lobule is another interesting finding and suggests the impairment of motor function. Physiologically, the paracentral lobule is an important component of the descending motor system, which passes through the brainstem (Hua et al., 2012; Lin et al., 2017). In RT of NPC, the brainstem is included in the radiation field and is exposed to high-dose radiation. We speculate that radiation-related brainstem injury may result in damage of the primary motor pathways, which induces functional abnormality in the paracentral lobule. Motor dysfunction is a common clinical symptom in patients with NPC following RT and has been supported by several neuroimaging studies (Lin et al., 2017; Zhang et al., 2020a). Our findings of paracentral lobule alterations in 2D fALFF provide further functional explanation for the impaired motor function observed in patients in the post-RT group.

### Classification Performance of Insular and STG Surface-Based Falff

In the SVM analysis, surface-based fALFF in the left insula and bilateral STG proved to have an impressive performance in identifying patients likely to develop RE. In contrast, surface-based fALFF in the more commonly affected brain regions (such as the temporal pole and the mesial temporal lobe) contributed little to the prediction of RE. These findings indicate that the radiation-induced differences in spontaneous neuronal activity in the temporal pole, being relatively slight, could be concealed by the more powerful ones in the insula and STG. However, the exact cause remains unknown. One possible explanation may be related to off-target effects (Beera et al., 2018) as evidenced by the fact that RT of the near-end brain regions (such as the temporal pole and the mesial temporal lobe) may result in off-target brain activity differences in far-end regions (such as the insula and STG) (Ding et al., 2018). In fact, widely functional or structural links between the temporal pole/mesial temporal lobe and insula, as well as the STG, have been well documented in previous studies (Nagai et al., 2007; Almashaikhi et al., 2014; Nachtergaele et al., 2019). A neuroimaging study reported that the temporal pole and mesial temporal lobe have close intrinsic functional connections with the insula and STG (Almashaikhi et al., 2014). A more recent anatomical study revealed a temporoinsular projection system tightly coupling the insula and temporal pole/mesial temporal lobe *via* white matter fibers from the extreme capsule and the uncinate fasciculus (Nachtergaele et al., 2019). Taken together, our SVM analysis findings suggest that off-target effects may play an important role in the prediction of RE in the pre-symptomatic stage.

### A Potential Imaging Biomarker for Radiation-Induced Brain Injury

In this study, we found that surface-based fALFF rather than volume-based fALFF could characterize radiation-induced functional alterations in patients in the post-RT group as evidenced by its effective detection of imperceptible cerebral changes and impressive classification performance in identifying patients who develop RE. Therefore, surface-based fALFF may be a novel imaging biomarker of RE. Although volume-based functional metrics have been commonly adopted to investigate functional alterations in a variety of diseases, they neglect the intersubject variability in cortical folding patterns (Li et al., 2014). In contrast, in the surface-based space, anatomical regions can be accurately matched with the same locations in the standard template, which decreases intersubject variability and increases statistical power (Fischl et al., 1999; Argall et al., 2006; Li et al., 2014; Zhang Y. et al., 2019). Therefore, compared with volume-based functional metrics, surface-based ones are more specific to the intrinsic functional organization of the cortical mantle and have moderate to high test–retest reliability (Jiang and Zuo, 2016). Two recent studies showed that surface-based ReHo, instead of volume-based ReHo, could be a useful index to investigate the pathogenesis of major depressive disorder and bipolar disorder (Li et al., 2014; Zhang Y. et al., 2019). Similar phenomena could also be observed in the structural domain. Several previous studies have reported that cortical thickness or cortical area is more sensitive than VBM in showing macro-neuroanatomical differences because VBM provides a mixed measure of cortical area and cortical thickness, whereas cortical thickness or cortical area provides a measure of specific pathological processes perpendicular or tangential to the cortical surface (Singh et al., 2006; Grant et al., 2015; Zhang et al., 2018). Considering that brain structure is the foundation of brain function, the superiority of surface-based structural indices in detecting morphological changes provides further evidence for the notion that a surface-based functional metric could be a potential imaging biomarker for a specific disease.

## Limitations

This study had several limitations. First, the diagnosis of RE was not confirmed histopathologically; it may not be ethically or medically proper to perform confirmatory brain biopsy considering the associated risks. Second, several confounding factors were present, such as varying TNM stage, chemotherapy regimens, and radiation fields. Third, detailed evaluations of the quality of life and cognitive function were not performed, which weakens the interpretability of our findings. Fourth, a relatively small sample size in this study may lower the statistical power and result in overfitting, although the SVM strategy was adopted to address this issue. In addition, this study is mainly neuroimaging oriented and the biomarker information from other domains, such as blood, is insufficient. Our findings should be confirmed by future multiscale longitudinal studies with large samples that additionally assess cognition.

## Conclusion

Our findings of surface-based fALFF alterations in intratemporal and extratemporal lobe locations and their impressive performance, in identifying patients likely to develop RE, suggest that surface-based fALFF may serve as a sensitive imaging biomarker to predict RE in patients with NPC who undergo RT.

## Data Availability Statement

The original contributions presented in the study are included in the article/Supplementary Material, further inquiries can be directed to the corresponding authors.

## Ethics Statement

The studies involving human participants were reviewed and approved by Xiangya hospital, Central South University. The patients/participants provided their written informed consent to participate in this study.

## Author Contributions

Y-mZ, LL, W-hL, and L-rS conceived and designed the experiments. Y-fK, Y-mZ, J-jZ, and J-mG analyzed the data. Y-mZ, LL, J-mG, L-zL, and Y-fK contributed to reagents/materials/analysis tools. Y-mZ, Y-fK, J-jZ, W-hL, and L-rS wrote the manuscript. All authors read and approved the final manuscript.

## Conflict of Interest

The authors declare that the research was conducted in the absence of any commercial or financial relationships that could be construed as a potential conflict of interest.

## Abbreviations

NPC: nasopharyngeal carcinoma
RT: radiotherapy
RE: radiation encephalopathy
fALFF: fractional amplitude of low-frequency fluctuation
AUC: area under the curve
CT: computerized tomography
MRI: magnetic resonance imaging
MRS: magnetic resonance spectroscopy
DTI: diffusion tensor imaging
ROI: region of interest
WM: white matter
GM: gray matter
VBM: voxel-based morphometry
SBM: surface-based morphometry
rsfMRI: resting-state functional MRI
ReHo: regional homogeneity
CNS: central nervous system
BOLD: blood oxygenation-level dependent
PCC: posterior cingulate cortex
MDRT: maximum dosage of radiation-treatment
3D: three-dimensional/volume-based/voxel-based
2D: two-dimensional/surface-based/vertex-based
UICC/AJCC: International Union against Cancer /American Joint Committee on Cancer
TNM: (T = Tumor, N = Nodes and M = Metastasis)
IMRT: Intensity-modulated radiation therapy
2D-CRT: conventional two-dimensional radiotherapy
KPS: Karnofsky Performance Status
MPRAGE: magnetization prepared rapid acquisition gradient echo
FOV: field of view
TE: echo time
TR: repetition time
BBR: boundary-based registration
SVM: support vector machine
LIBSVM: A Library for Support Vector Machines
LOOCV: leave-one-out cross-validation
SD: standard deviation
PT: permutation test
TFCE: threshold-free cluster enhancement
FWE: family-wise error
DPABI: Data Processing & Analysis for (Resting-State) Brain Imaging
ITG: inferior temporal gyrus
DLPFC: dorsolateral prefrontal cortex
MPFC: mesial prefrontal cortex
STG: superior temporal gyrus
ECN: executive control network
SN: salience network.
