# Supplementary material for: Surface-Based Falff: A Potential Novel Biomarker for Prediction of Radiation Encephalopathy in Patients With Nasopharyngeal Carcinoma
Source: Front Neurosci. 2021 Jul 19;15:692575. doi: 10.3389/fnins.2021.692575 (PMC8326829; doi:10.3389/fnins.2021.692575)
Supplement: Supplementary file 1 [file Table_1.pdf]

Supplementary materials

S1. Diagnostic criteria for RE

Three important aspects (including a medical history of radiotherapy, typical MRI features, and exclusion of other possible differential diagnosis) should be considered in the diagnosis of RE (**Figure S1**). The typical MRI features were mainly including: (1) Dotted, patchy inhomogeneous lesions in gray matter and whiter matter of unilateral or bilateral temporal lobes, with slight hyper-intensity and hypo-intensity on T2- and T1-weighted images respectively; (2) Peripheral edema surrounding the lesions in temporal lobes showing as finger-shaped high signal intensity areas on T2-weighted images； (3) Cystic degeneration with round or oval shape and well-defined margin. 4) Nodular hemosiderin deposition with hypo-intense alterations on both T1- and T2-weighted images. Together with the dynamic MRI manifestations and clinical or laboratory evidence, patients with cerebral infarction, abscess, brain tumor, or NPC's intracranial invasion were excluded from this study (Chan et al. 1999, Tang et al. 2012, Zhou et al. 2017, Zhang et al. 2019).

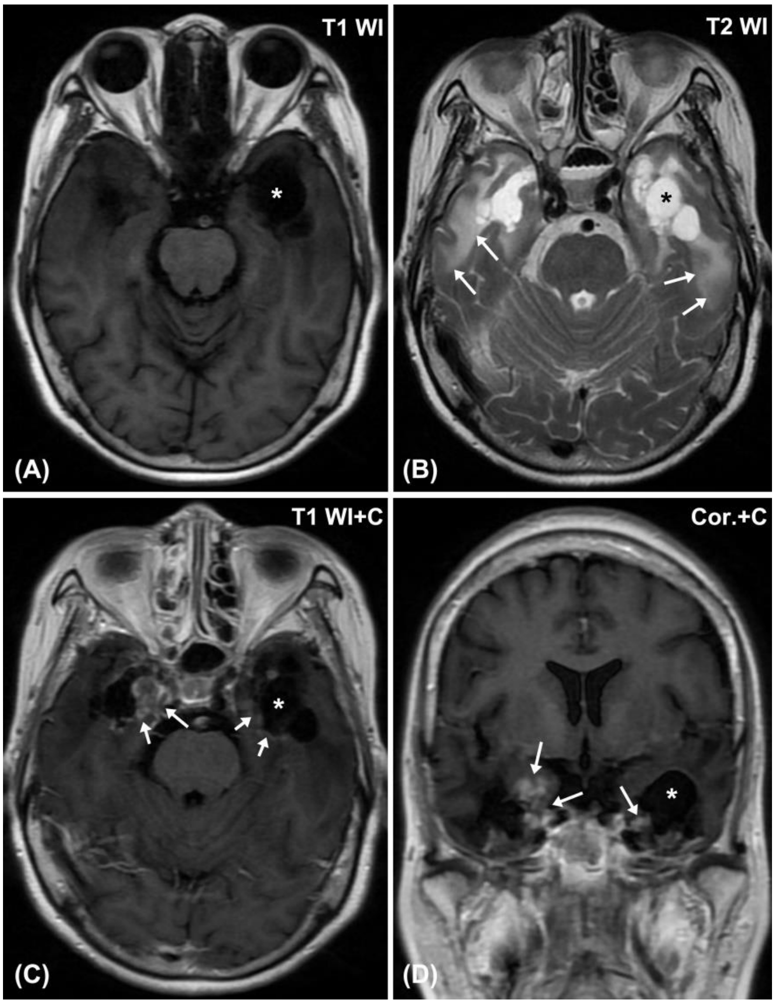

Figure S1

Figure S1: MRI findings of a 61-year-old woman with RE. Patchy inhomogeneous lesions with hypo-intensity on T1 WI (A, asterisk), hyper-intensity on T2 WI (B, asterisk) and surrounding slight hyper-intensity areas (B, arrows) are observed in bilateral temporal lobes. The lesions displays heterogeneous edged enhancement (C and D, arrows) with central necrosis and cystic degeneration (C and D, asterisks).

S2. Detailed information on radiotherapy and chemotherapy for NPC patients

All the patients in the Post-RT group were treated with intensity-modulated radiation therapy (IMRT) (Zhang et al. 2015) or conventional two-dimensional radiotherapy

(2D-CRT) (Lai et al. 2011). Specifically, 15 and 6 patients in the Post-RT $_{\text{non-RE}}$ group, as well as 12 and 6 patients in the Post-RT RE $_{\text{proved in follow-up}}$ group were treated by IMRT and 2D-CRT respectively (**Table 1**). As previously reported (Lin et al. 2017, Zhang et al. 2018), for patients treated with IMRT, the primary nasopharyngeal tumor and the upper neck above the inferior margin of the cricoid cartilage were covered. Inverse IMRT planning and a MIMiC multi-leaf collimator (Nomos, Sewickley, PA, United States) were adopted to make a treatment plan. The total dose of RT was 58–70 Gy, which was divided into 30–33 fractions (Lin et al. 2017, Zhang et al. 2018). The patients were treated with 1 fraction daily over 5 days per week. For 2D-CRT treatment, two lateral opposing faciocervical portals were used to irradiate the nasopharynx and the upper neck in one volume (Zhang et al. 2018). The shrinkingfield technique was then adopted to limit the irradiation of the spinal cord. An accumulated radiation dose of 66–76 Gy was applied to the primary tumor for each patient. Concurrent chemoradiotherapy with/without neoadjuvant/adjuvant chemotherapy were recommended for patients staged IIb to IVa–b at 1–3 months before/after RT, with one or more chemotherapeutics, such as cisplatin, paclitaxel, nedaplatin and fluorouracil (Zhang et al. 2018, Zhang et al. 2019).

References

Chan, Y. L., Leung, S. F., King, A. D., Choi, P. H. and Metreweli, C. (1999). Late radiation injury to the temporal lobes: morphologic evaluation at MR imaging. *Radiology* 213, 800-807.doi: 10.1148/radiology.213.3.r99dc07800

Lai, S. Z., Li, W. F., Chen, L., Luo, W., Chen, Y. Y., Liu, L. Z., et al. (2011). How does intensity-modulated radiotherapy versus conventional two-dimensional radiotherapy influence the treatment results in nasopharyngeal carcinoma patients? *Int J Radiat Oncol Biol Phys* 80, 661-668.doi: 10.1016/j.ijrobp.2010.03.024

Lin, J., Lv, X., Niu, M., Liu, L., Chen, J., Xie, F., et al. (2017). Radiation-induced abnormal cortical thickness in patients with nasopharyngeal carcinoma after radiotherapy. *Neuroimage Clin* 14, 610-621.doi: 10.1016/j.nicl.2017.02.025

Tang, Y., Luo, D., Rong, X., Shi, X. and Peng, Y. (2012). Psychological disorders, cognitive dysfunction and quality of life in nasopharyngeal carcinoma patients with radiation-induced brain injury. *PLoS One* 7, e36529.doi: 10.1371/journal.pone.0036529

Zhang, M. X., Li, J., Shen, G. P., Zou, X., Xu, J. J., Jiang, R., et al. (2015). Intensity-modulated radiotherapy prolongs the survival of patients with nasopharyngeal carcinoma compared with conventional two-dimensional radiotherapy: A 10-year experience with a large cohort and long follow-up. *Eur J Cancer* 51, 2587-2595.doi: 10.1016/j.ejca.2015.08.006

Zhang, Y., Yi, X., Gao, J., Li, L., Liu, L., Qiu, T., et al. (2019). Chemotherapy Potentially Facilitates the Occurrence of Radiation Encephalopathy in Patients With Nasopharyngeal Carcinoma Following Radiotherapy: A Multiparametric Magnetic Resonance Imaging Study. *Front Oncol* 9, 567.doi: 10.3389/fonc.2019.00567

Zhang, Y. M., Chen, M. N., Yi, X. P., Li, L., Gao, J. M., Zhang, J. L., et al. (2018). Cortical Surface Area Rather Than Cortical Thickness Potentially Differentiates Radiation Encephalopathy at Early Stage in Patients With Nasopharyngeal Carcinoma. *Front Neurosci* 12, 599.doi: 10.3389/fnins.2018.00599

Zhou, X., Liao, X., Ren, X., Xiang, K., Hu, Q., Zhang, M., et al. (2017). Dynamic MRI follow-up of radiation encephalopathy in the temporal lobe following nasopharyngeal carcinoma radiotherapy. *Oncol Lett* 14, 715-724.doi: 10.3892/ol.2017.6199

S3. FMRI data preprocessing for volume-based fALFF

The volume-based (3D) fMRI data were preprocessed using a module named "Data Processing Assistant for Resting State fMRI Advanced Edition" (DPARSFA) in DPABI (Data Processing & Analysis for Brain Imaging) software (http://rfmri.org/dpabi) (Yan et al. 2016, Neuroinformatics). The first 10 time points of each scan were discarded to minimize the confounding effects of magnetic field instability. Then, several preprocessing steps including slice timing, realign, spatial normalization, resampling to 3mm × 3mm × 3 mm voxel size, nuisance signals regression (including head motion parameters, white matter, cerebrospinal fluid, and global mean signals), and spatial smoothing (with a 4-mm Gaussian kernel) were subsequently conducted. A fALFF was then calculated for each participant (for details of fALFF calculation, see Zou et al. 2008, J Neurosci Methods). After that, temporally bandpass filtered (0.01–0.1 Hz) for time-series in each voxel were performed.

References

Yan, C.G., Wang, X.D., Zuo, X.N., Zang. Y.F. (2016). DPABI: Data Processing & Analysis for (Resting-State) Brain Imaging. *Neuroinformatics* 14, 339-351. doi：10.1007/s12021-016-9299-4.

Zou, Q.H., Zhu, C.Z., Yang, Y.H., Zuo, X.N., Long, X.Y., Cao, Q.J., et al. (2008). An improved approach to detection of amplitude of low-frequency fluctuation (ALFF) for resting-state fMRI: fractional ALFF. *J NEUROSCI METH* 172, 137-141. doi: 10.1016/j.jneumeth.2008.04.012

S4. Supplementary tables

Table S1. Clusters showing group surface-based fALFF differences between Post-RT $_{\text{non-RE}}$ and Pre-RT

**Brain regions**	**MNI coordinates**			**Hemisphere**	**Cluster size (mm^2)**	**Peak T values**	**P value***
	**X**	**Y**	**Z**				
Insular cortex, SII	-36	-34	20	L	1505	-5.33	<0.05
Cingulate, paracentral lobule	-11	7	39	L	150	-5.32	<0.05
Lateral Temporal Cortex, inferior temporal sulcus and gyrus	-40	-48	-18	L	646	4.57	<0.05
Medial Prefrontal Cortex, dorsolateral prefrontal cortex	-19	51	-14	L	935	4.37	<0.05

Note: fALFF, fractional amplitude of low-frequency fluctuation; SII, second somatosensory area, MNI Montreal Neurological Institute; L left. * PT-TFCE-FWE corrected.

Table S2. Clusters showing group surface-based fALFF differences between Post-RT $_{\text{RE proved in follow-up}}$ and Pre-RT

Brain regions	MNI coordinates			Hemisphere	Cluster size (mm^2)	Peak T values	P value*
	X	Y	Z				
FFC, hippocampus and parahippocampal gyrus	-40	-48	-19	L	713	5.85	<0.05
Visual Cortex, including V1 and V2	-5	-94	-9	L	56	4.33	<0.05

Note: fALFF, fractional amplitude of low-frequency fluctuation; FFC, Fusiform Face Complex; MNI Montreal Neurological Institute; L left. * PT-TFCE-FWE corrected.

Table S3. Clusters showing group surface-based fALFF differences between Post-RT $_{\text{RE proved in follow-up}}$ and Post-RT $_{\text{non-RE}}$

Brain regions	MNI coordinates			Hemisphere	Cluster size (mm^2)	Peak T values	P value*
	X	Y	Z				
Insular cortex	0.59	-11	19	R	250	5.8	<0.05

Note: fALFF, fractional amplitude of low-frequency fluctuation; MNI Montreal Neurological Institute; R right. * PT-TFCE-FWE corrected.
